# Inflammatory and Hypercoagulable Biomarkers and Clinical Outcomes in COVID-19 Patients

**DOI:** 10.3390/jcm10143086

**Published:** 2021-07-13

**Authors:** Hiroki Kitakata, Shun Kohsaka, Shunsuke Kuroda, Akihiro Nomura, Takeshi Kitai, Taishi Yonetsu, Sho Torii, Yuya Matsue, Shingo Matsumoto

**Affiliations:** 1 Department of Cardiology, Keio University School of Medicine, Tokyo 160-8582, Japan; kitkat@keio.jp; 2 Department of Cardiovascular Medicine, Cleveland Clinic, Cleveland, OH 44195, USA; kuroshun113@gmail.com; 3 Department of Cardiovascular Medicine, Kanazawa University Graduate School of Medical Sciences, Kanazawa 920-8641, Japan; anomura@med.kanazawa-u.ac.jp; 4 Department of Cardiovascular Medicine, Kobe City Medical Center General Hospital, Kobe 650-0047, Japan; t-kitai@kcho.jp; 5 Department of Interventional Cardiology, Tokyo Medical and Dental University, Tokyo 113-8513, Japan; yonetsu@gmail.com; 6 Department of Cardiology, Tokai University School of Medicine, Isehara 259-1193, Japan; shoz3333@gmail.com; 7 Department of Cardiovascular Biology and Medicine, Juntendo University Graduate School of Medicine, Tokyo 113-8421, Japan; yuya8950@gmail.com; 8 Cardiovascular Respiratory Sleep Medicine, Juntendo University Graduate School of Medicine, Tokyo 113-8421, Japan; 9 Department of Cardiovascular Medicine, Department of Internal Medicine, Toho University Faculty of Medicine, Tokyo 143-8540, Japan; shingomatsumoto0606@gmail.com

**Keywords:** COVID-19, biomarker, CRP, D-dimer, cardiovascular disease

## Abstract

Systemic inflammation and hypercoagulopathy are known pathophysiological processes of coronavirus disease 2019 (COVID-19), particularly in patients with known cardiovascular disease or its risk factors (CVD). However, whether a cumulative assessment of these biomarkers at admission could contribute to the prediction of in-hospital outcomes remains unknown. The CLAVIS-COVID registry was a Japanese nationwide retrospective multicenter observational study, supported by the Japanese Circulation Society. Consecutive hospitalized patients with pre-existing CVD and COVID-19 were enrolled. Patients were stratified by the tertiles of CRP and D-dimer values at the time of admission. Multivariable Cox proportional hazard models were constructed. In 461 patients (65.5% male; median age, 70.0), the median baseline CRP and D-dimer was 58.3 (interquartile range, 18.2–116.0) mg/L and 1.5 (interquartile range, 0.8–3.0) mg/L, respectively. Overall, the in-hospital mortality rate was 16.5%, and the rates steadily increased in concordance with both CRP (5.0%, 15.0%, and 28.2%, respectively *p* < 0.001) and D-dimer values (6.8%, 19.6%, and 22.5%, respectively *p* = 0.001). Patients with the lowest tertiles of both biomarkers (CRP, 29.0 mg/L; D-dimer, 1.00 mg/L) were at extremely low risk of in-hospital mortality (0% until day 50, and 1.4% overall). Conversely, the elevation of both CRP and D-dimer levels was a significant predictor of in-hospital mortality (Hazard ratio, 2.97; 95% confidence interval, 1.57–5.60). A similar trend was observed when the biomarker threshold was set at a clinically relevant threshold. In conclusion, the combination of these abnormalities may provide a framework for rapid risk estimation for in-hospital COVID-19 patients with CVD.

## 1. Introduction

Coronavirus disease 2019 (COVID-19) is a respiratory disease caused by severe acute respiratory syndrome coronavirus 2 (SARS-CoV-2) [1]. As of 14 February 2021, the total number of confirmed cases reached over 100 million, and the number of global deaths was estimated to be over 2 million people [2]. While pre-existing cardiovascular disease or its risk factors (CVD) have been reportedly associated with poor outcomes in patients with COVID-19 [3,4,5,6,7], further identification of risk factors for poor outcomes among these subjects is not well known.

Recent investigations have demonstrated that moderate or severe COVID-19 is characterized by an intense inflammatory syndrome associated with hypercoagulopathy [8,9]. Both of these pathophysiological processes have known representative biomarkers: C-reactive protein (CRP) and D-dimer. Several studies have described the potential role of biomarkers in evaluating the severity of COVID-19 [10]. Liu et al. showed that serum levels of CRP and IL-6 could effectively predict clinical severity in patients with COVID-19 [11]. Another study also reported that the initial CRP level on admission was an independent predictor of severe or critical illness in COVID-19 patients [12]. More recently, Zhang et al. reported that D-dimer level on admission could effectively predict in-hospital mortality in patients with COVID-19 [13]. However, most of the studies were conducted in a single hospital and were relatively small, which precludes the generalizability of their results.

The clinical outcomes of COVID-19 infection in hospitalized patients with cardiovascular disease and/or risk factors (CLAVIS-COVID) was a Japanese nationwide retrospective multicenter observational study supported by the Japanese Circulation Society. This study was designed to elucidate the outcomes of COVID-19 infection among patients with pre-existing CVD. In Japan, biomarkers are routinely measured as the associated cost is universally covered, providing us the unique opportunity for their assessment.

Here, we evaluated whether a cumulative assessment of biomarkers such as CRP and D-dimer levels at admission and their dynamics would provide prognostic information for risk stratification in COVID-19 patients with pre-existing CVD.

## 2. Methods

Data that support the findings of this study are available from the corresponding author upon reasonable request.

### 2.1. Study Design and Patient Population

CLAVIS-COVID is a case-control study that included 693 consecutive patients with pre-existing CVD and COVID-19 who were admitted to 49 nationwide Japanese acute care hospitals between 1 January 2020 and 31 May 2020. The infection of COVID-19 was diagnosed on the basis of a positive polymerase chain reaction (PCR) result from nasal or pharyngeal swabs in all patients.

In the present study, the main population consisted of patients with pre-existing CVD or their risk factors (hypertension, diabetes mellitus, or dyslipidemia). Pre-existing CVD was defined as history and/or manifestations of heart failure, coronary artery disease, myocardial infarction, peripheral artery disease, valvular heart disease, cardiac arrhythmia, pericarditis, myocarditis, congenital heart disease, pulmonary hypertension, deep vein thrombosis, pulmonary embolism, aortic dissection, aortic aneurysm, cerebral infarction/transient ischemic attack, the use of cardiac devices (pacemaker, implantable cardioverter defibrillation, cardiac resynchronization therapy, and left ventricular assist device), heart transplantation, and cardiac arrest. Detailed definitions of each comorbidity have been described in the literature [14]. Patients who were under 20 years of age at admission were excluded from this study.

The study protocol conformed to the ethical guidelines of the 1975 Declaration of Helsinki. The study protocol, including the use of an opt-out consent method, was approved by the ethics committee of Toho University Omori Medical Center (no. M20253), and each of the ethics committees of all participating institutions. This clinical study was registered with the University Hospital Medical Information Network Clinical Trial Registry, in accordance with the International Committee of Medical Journal Editors (UMIN-ID: UMIN000040598).

### 2.2. Data Collection

Our dataset referred to the case report form proposed by the International Severe Acute Respiratory and Emerging Infection Consortium [15]. The collected baseline clinical information, which included symptoms, demographics, medical history, home medications, baseline comorbidities, physical findings, laboratory tests, X-ray and chest CT findings, electrocardiogram and cardiac echocardiography results, treatment information, and outcomes, were obtained from electronic medical records using data collection forms. Additionally, all laboratory and imaging data were collected at the time of admission, which was considered the date of COVID-19 onset if the patient presented with symptoms of COVID-19. In the case that the patient had no symptoms on admission, the date of onset was defined as the day of the first positive PCR test.

### 2.3. Decision of Hospitalization and Discharge in Patients with COVID-19

During the period of patient enrollment, between 1 January 2020 and 31 May 2020, the Japanese government mandated the hospitalization of all patients diagnosed with COVID-19 through PCR testing, regardless of their severity. The hospitals participating in the current study fundamentally followed this government recommendation. The triaging and discharge thresholds of all COVID-19 patients were pre-defined according to the Japanese government guidelines for COVID-19 management [16]. To prevent a shortage of hospital beds, the Japanese health ministry notified municipalities to have COVID-19 patients with mild or no symptoms stay at accommodation facilities. The ministry guidelines encourage the patient to stay in-hospital until (1) systemic conditions and respiratory symptoms are stable, (2) body temperature is consistently under 37.5 °C for at least 24 h, and (3) negative PCR results are confirmed twice, at least 12 h apart.

### 2.4. Biomarker Measurement

The assays for CRP and D-dimer were performed in the laboratory of each study site. The assays included latex agglutination and latex turbidimetry (Supplemental Table S1A,B). Data on laboratory values for these biomarkers were reviewed, and the maximum and final levels prior to discharge for each biomarker were recorded. Patients were categorized by the tertiles of CRP (C1, <29.0 mg/L; C2, ≥29.0 to <92.0 mg/L; C3, ≥92.0 mg/L) and D-dimer (D1, <1.00 mg/L; D2, ≥1.00 to <2.28 mg/L; D3, ≥2.28 mg/L) concentrations at the timing of admission. The threshold was set on the values from the lowest tertile (CRP, 29.0 mg/L; D-dimer, 1.00 mg/L), and a cumulative incidence of the primary outcome was analyzed (“both values above threshold” vs. “either of the values above threshold” vs. “neither value above threshold”). For sensitivity analyses, we set three additional cut-off values, and the incidence of in-hospital death was evaluated: (1) Thresholds from the highest tertiles (CRP, 92.0 mg/L; D-dimer, 2.28 mg/L); (2) thresholds from the clinically relevant cut-off values (CRP, 50.0 mg/L; D-dimer, 1.00 mg/L) [17,18,19,20], and (3) thresholds calculated by ROC analysis for each biomarker.

### 2.5. Study Outcome

All patients enrolled in this study had in-hospital information available until 8 November 2020, the deadline of data transfer. The primary outcome of this study was all-cause in-hospital mortality. The secondary outcomes were the major cardiovascular events during admission. Major cardiovascular events were defined as the composite of stroke, coronary artery disease including myocardial infarction, acute decompensated heart failure, the incidence of moderate or severe valvular heart disease, myocarditis, embolism in large vessels, aortic dissection, aortic aneurysm, and peripheral artery disease. Designated data entry operators and data managers in each institution were able to access the REDCap [21] website to register and edit the case data. The credibility of the data was maintained systematically through a checking system by cardiologists in each institution. Moreover, the quality of the reporting was verified by the core investigator (Shunsuke Kuroda), and queries were conducted to ensure quality.

### 2.6. Statistical Analysis

Continuous variables were expressed as mean ± standard deviation for normally distributed variables and median with interquartile range (IQR) for non-normally distributed variables. Categorical variables were expressed as percentages. Student’s *t*-test or Mann–Whitney’s *U* test was used to compare normally or non-normally distributed variables and Pearson’s chi-squared test was used to compare categorical variables. Differences in CRP and D-dimer values by group were compared using repeated-measures analysis of variance (ANOVA). The Cox proportional hazard model was used to estimate the odds of in-hospital mortality, adjusted for demographics and clinical comorbidities. Covariates included in the multivariable models were age, sex, body mass index, hypertension, diabetes mellitus, hyperlipidemia, coronary artery disease, previous history of cancer, chronic obstructive pulmonary disease, chronic kidney disease, and baseline laboratory values. Furthermore, for another model, we analyzed all clinical and laboratory parameters for their prognostic value in univariate analysis and include only variables with *p* < 0.1 in multivariate analysis. Cumulative incidence and adjusted odds of the primary outcome were analyzed to evaluate the combination of CRP and D-dimer levels (“both above threshold” vs. “either of the values above threshold” vs. “neither above threshold”). The Kaplan–Meier method was used to evaluate the impact of the initial CRP and D-dimer levels on subsequent in-hospital death and was compared using the log-rank statistic. We also performed sensitivity analysis based on the clinical threshold provided in the Biomarker Measurement section. For all statistical analyses, significance was defined at a *p* value of <0.05. Data were analyzed using SPSS version 26 (IBM Corp., Armonk, NY, USA).

## 3. Results

**Figure 1:**
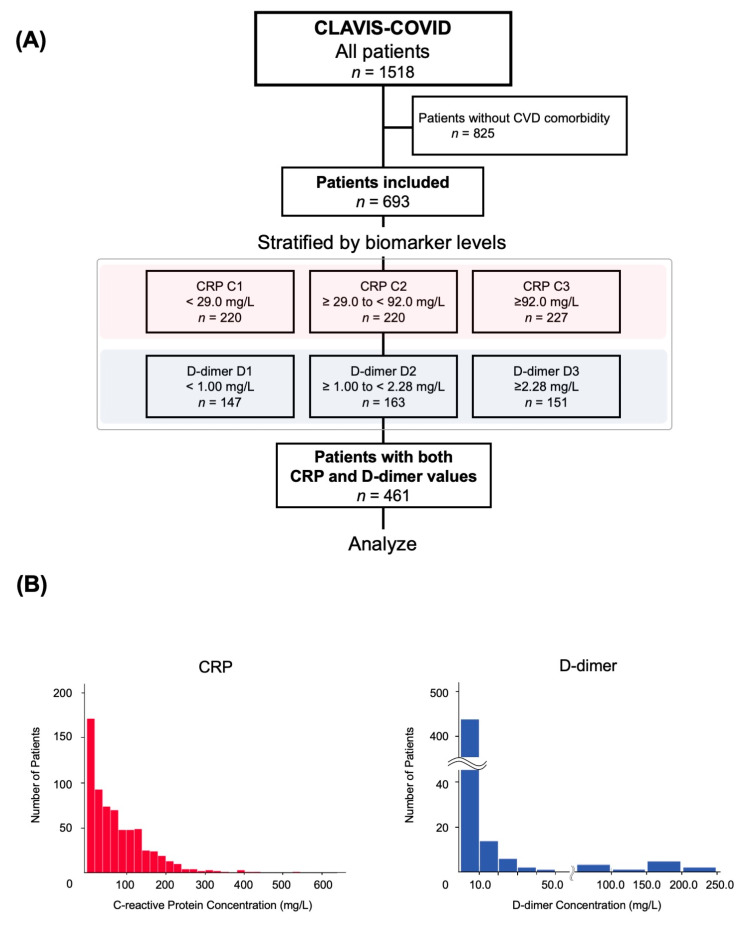
Flowchart describing the study design from the CLAVIS-COVID registry (**A**), and patient distribution of initial CRP and D-dimer concentrations (**B**). CVD: Cardiovascular medical condition, CRP: C-reactive protein.

First, a total of 693 patients with a history of CVD (64.8% male; median age, 70.0 years old) were analyzed (Figure 1A). Among them, the proportion of Japanese patients was 96.1%, and the median length of hospital stay was 18 (IQR, 11–29) days. CRP and D-dimer levels were measured at the time of admission in 667 (96.2%) and 461 (66.5%) patients, respectively. The median baselines were 58.3 (IQR, 18.2–116.0) mg/L and 1.5 (IQR, 0.8–3.0) mg/L, respectively.

**Figure 2:**
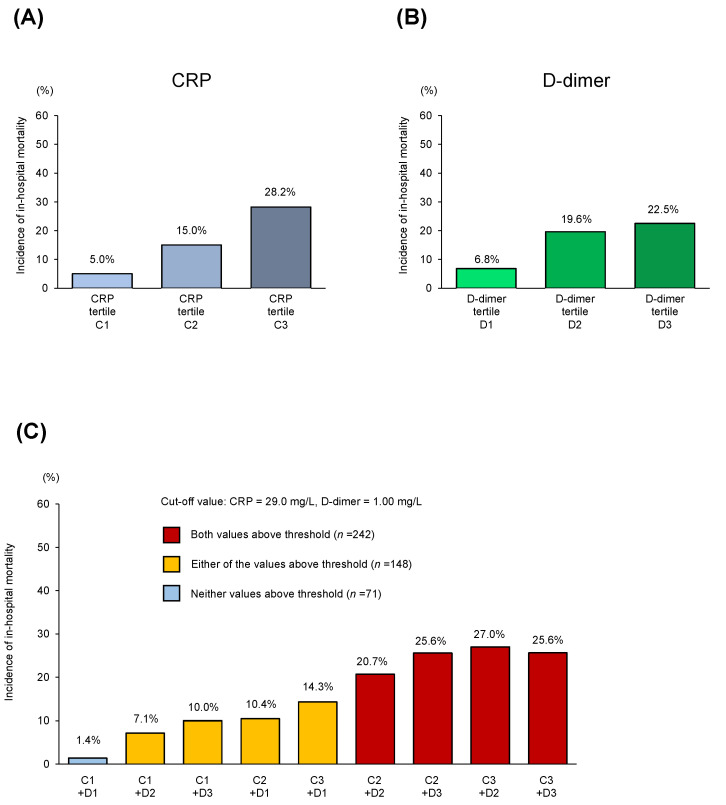
Mortality rate (%) among patients with pre-existing CVD and COVID-19 infection, stratified by initial CRP (**A**) or D-dimer (**B**) concentrations. Patients were categorized by the tertiles of CRP (C1, <29.0 mg/L; C2, ≥29.0 to <92.0 mg/L; C3, ≥92.0 mg/L) or D-dimer (D1, <1.00 mg/L; D2, ≥1.00 to <2.28 mg/L; D3, ≥2.28 mg/L) concentration at the timing of admission. (**C**) The cumulative incidence of in-hospital mortality, stratified by “both values above threshold”, “either of the values above threshold”, and “neither value above threshold”. CVD: Cardiovascular medical condition, COVID-19: Coronavirus disease 2019, CRP: C-reactive protein.

The distribution of CRP and D-dimer levels is shown in Figure 1B. Among the 461 patients with initial values of both CRP and D-dimer, in-hospital mortality was 16.5% (*n* = 76). Figure 2 demonstrates the in-hospital mortality among the patients in this study stratified by the initial CRP (Figure 2A) and D-dimer levels (Figure 2B). In-hospital death was concordantly associated with baseline CRP values (C1, 5.0%; C2, 15.0%; C3, 28.2% [*p* < 0.001]) and D-dimer values (D1, 6.8%; D2, 19.6%; D3, 22.5% [*p* = 0.001]).

**Figure 3:**
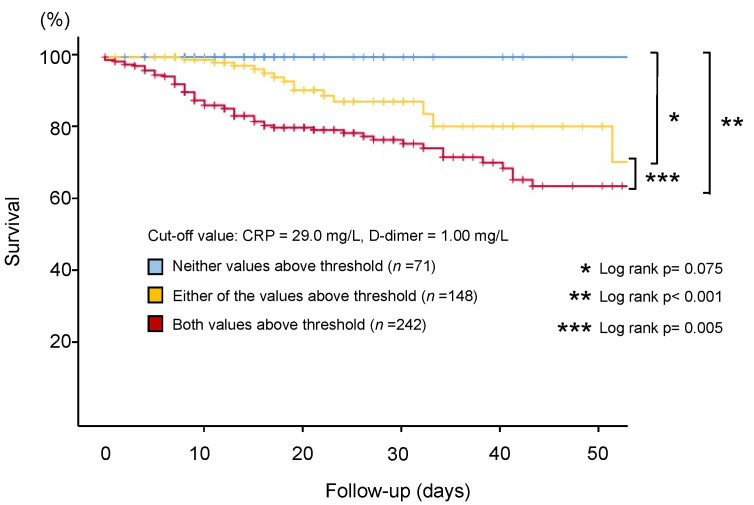
Kaplan–Meier analysis demonstrating the impact of the initial CRP and D-dimer level on subsequent in-hospital death. Threshold values were CRP, 29.0 mg/L; D-dimer, 1.00 mg/L. CRP: C-reactive protein.

Patients with initial values of both CRP and D-dimer (*n* = 461) were divided into three groups (“both values above threshold” vs. “either of the values above threshold” vs. “neither value above threshold”; Table 1) with a cut-off value (CRP, 29.0 mg/L; D-dimer, 1.00 mg/L) for a cumulative analysis. The cumulative evaluation demonstrated that patients in the “neither value above threshold” group were at the lowest risk for in-hospital mortality (1.4%; Figure 2C). Kaplan–Meier estimates demonstrated that the “Both” (both values above threshold) group showed significantly higher crude rates of in-hospital mortality compared with the patients in the “Either” (either of the values above threshold) or the “Neither” (neither value above threshold) groups (Both vs. Either, *p* = 0.005; Both vs. Neither, *p* < 0.001; Figure 3). Notably, patients in the “Neither” group showed no in-hospital deaths within 50 days of hospitalization. Univariate cox hazard proportional analysis demonstrated that the elevation of both biomarkers was an independent risk factor for in-hospital mortality (hazard ratio (HR), 3.46; 95% confidence interval (CI, 1.72–6.96)), while the single elevation of the D-dimer was not (HR, 1.00; 95% CI, 1.00–1.01; Supplemental Table S2). After adjustment, the elevation of both biomarkers remained an independent risk factor for in-hospital mortality (HR, 2.97; 95% CI, 1.57–5.60; Table 2). Another model also demonstrated that the elevation of both biomarkers remained an independent risk factor for in-hospital mortality (HR, 3.88; 95% CI, 1.19–12.7; Supplemental Table S3).

**Figure 4:**
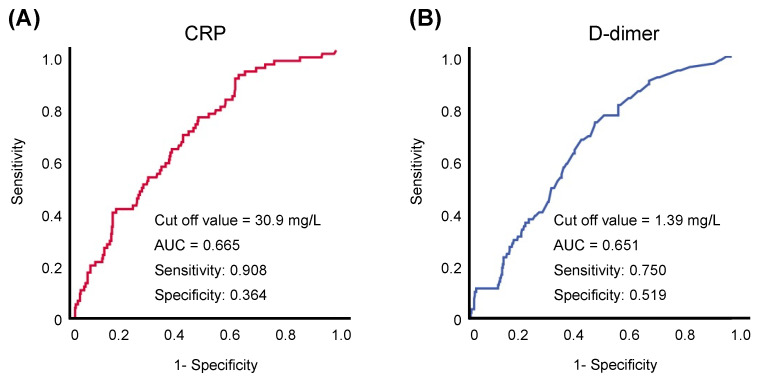
ROC analysis demonstrating the predictive threshold values of (**A**) CRP and (**B**) D-dimer for predicting in-hospital death. Threshold values were CRP, 30.9 mg/L; D-dimer, 1.39 mg/L. CRP: C-reactive protein. AUC: Area under the curve.

**Figure 5:**
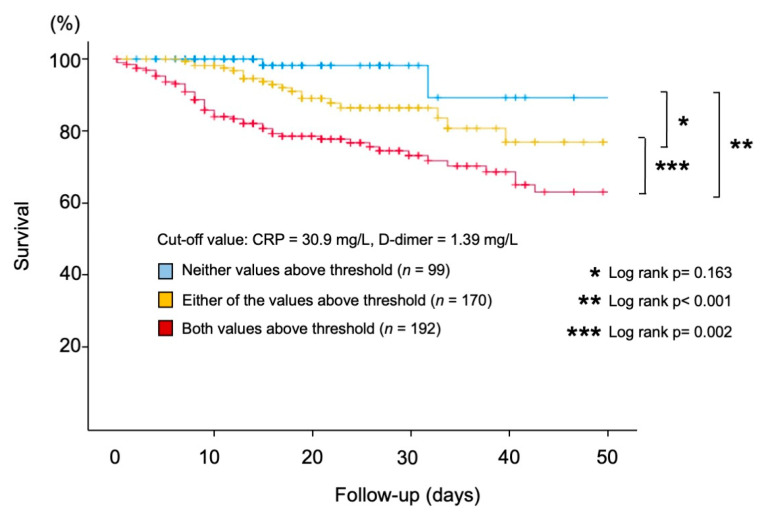
Kaplan–Meier analysis demonstrating the impact of the initial CRP and D-dimer level on subsequent in-hospital death. Threshold values were based on ROC analysis: CRP, 30.9 mg/L; D-dimer, 1.39 mg/L. CRP: C-reactive protein.

Supplemental Figure S1A demonstrates the outcomes when a threshold is set on the second cut-off value on each tertile (CRP, 92.0 mg/L; D-dimer, 2.28 mg/L). Kaplan–Meier analysis demonstrated that the “Both” group showed significantly higher crude rates of in-hospital mortality compared with the patients in the “Neither” group (Both vs. Neither; *p* = 0.024; Supplemental Figure S1A). Furthermore, even when we set the cut-off value along with previous literature (CRP, 50.0 mg/L; D-dimer, 1.00 mg/L), the “Both” group still had higher crude rates of in-hospital mortality compared with the patients in the “Neither” group (Both vs. Either, *p* = 0.119; Both vs. Neither; *p* < 0.001; Supplemental Figure S1B). In addition, the ROC analysis demonstrated that the threshold for CRP and D-dimer were 30.9 mg/L and 1.39 mg/L, respectively (Figure 4A,B). When we set the cut-off value based on this analysis, the “Both” group still had higher crude rates of in-hospital mortality compared with the patients in the “Neither” group (Both vs. Either, *p* = 0.002; Both vs. Neither; *p* < 0.001; Figure 5).

Supplemental Table S4 summarizes the initial, maximum, and final (prior to discharge) values of each biomarker stratified by the in-hospital death group and survival group. During their hospital stay, most of the patients experienced a steady increase in biomarker levels, regardless of in-hospital outcomes. However, the increment between baseline and peak values were higher in deceased patients compared to survivors (CRP, 117 mg/L vs. 0.15 mg/L (*p* < 0.001); D-dimer, 6.05 mg/L vs. 0.00 mg/L (*p* < 0.001); Supplemental Table S5). Furthermore, the timing of peak values of both biomarkers in the in-hospital group was significantly proximal to the discharge timing (i.e., delayed from admission; Supplemental Table S6). Supplemental Figure S2 demonstrates the proportion of patients whose peak biomarker values were recorded at the indicated time points (days from admission). In particular, while almost half of the patients in the survival group showed the highest CRP values on day 0 (i.e., at the time of admission), approximately 80% of patients in the in-hospital death group were detected after day 1. The measurement of other biomarkers is also shown as a comparison of CRP and D-dimer dynamics (Supplemental Figure S3A–E).

## 4. Discussion

In our study, the following key points were demonstrated. First, baseline CRP and D-dimer values were both associated with in-hospital mortality, even in patients with pre-existing CVD. Second, the combination of baseline CRP and D-dimer levels can predict in-hospital mortality. Finally, our study highlighted that patients with initial values of CRP (<29.0 mg/L) and D-dimer (<1.00 mg/L) showed the lowest in-hospital death during the 50 days of hospitalization.

CVD and its risk factors have been repeatedly demonstrated as major predictors of adverse outcomes in COVID-19 patients [3,4,5,22]. However, as CVD risk factors include a wide range of conditions (hypertension, diabetes, and hyperlipidemia), a considerable number of patients with COVID-19 meet this definition [5]. Therefore, further identification of risk factors for poor outcomes among patients with CVD crucial in improving disease management and patient outcomes. A number of recent studies have demonstrated that CRP and D-dimer levels are associated with the severity of COVID-19 symptoms and prolonged hospital stay [8,9,12,19,23,24,25,26,27]. In a multicenter prospective observational cohort study from Europe, Knight et al. demonstrated that CRP was an essential biomarker for risk stratification in COVID-19 [28]. Moreover, Smilowitz et al. demonstrated that CRP was strongly associated with critical illness and mortality [29]. In their study, they analyzed the initial CRP value among approximately 2500 patients who were hospitalized for COVID-19 infection during a pandemic period in New York. Our study adds that the clinical impact of CRP levels could be further strengthened by combining the results of D-dimer level measurements. In particular, the negative predictive value of the combination approach was substantial in our study, demonstrating no mortality within 50 days when neither biomarker was elevated. In line with our results, Spanish investigators recently provided a protocol for outpatient management for patients with COVID-19, stating that CRP and D-dimer levels could extract groups with very low rates of admission and no mortality [30]. 

We set our threshold based on tertiles considering the applicability of the study results (cut-off values of these biomarkers need to be set according to the distribution of the population). Although we were able to provide directionally similar results with absolute values set from previous clinical studies, regional disparities and differences in cut-off values are important aspects for interpreting the outcome of the present study. Significant differences in fatalities between Europe and Asia due to COVID-19 have been documented [31]. In our study, the highest tertile of CRP value (92.0 mg/L) was similar to the cut-off value defined by Smilowitz et al. (108 mg/L) [29]. The proportion of in-hospital mortality among patients in their cohort was approximately equivalent to that in our study (32.2% vs. 28.8%). Another study also demonstrated that the in-hospital mortality rate was 29.2% (33/113 patients) among COVID-19 patients with a CRP level of >100 mg/L [32]. Finally, a large-scale study using mathematical models also concluded that, when appropriately adjusted, the prevalence of mortality is quite similar across eight countries, including Europe, the United States, and East Asia [33]. Our findings underscore the multiple-step approach for biomarker interpretation in predicting the prognosis of COVID-19 [29,34,35]. 

Another strength of this study is that we elucidated not only the association between initial biomarker levels and clinical outcomes, but also unveiled transient changes along with the outcomes. Our results demonstrated that the peak value of biomarker timing was significantly proximal to the discharge timing in the in-hospital death group compared to in the survivor group, which was consistent with previous literature [36]. The findings of our study have significant clinical implications in that elevated CRP values during admission could be a sign of subsequent worse prognosis in patients with COVID-19 and pre-existing CVD. Indeed, while almost half of the patients in the survival group showed that their initial CRP values were the highest, the CRP values were further increased among approximately 80% of patients in the in-hospital death group. Furthermore, the increments between baseline and peak values of both CRP and D-dimer were higher in the in-hospital death group than in the survivor group. These tendencies were also detected in other biomarkers, such as ferritin and procalcitonin. Given that CRP and D-dimer require a minimal number of resources and are commonly tested in medical hospitals [29,37,38], it is reasonable to routinely follow these biomarkers to predict the clinical trajectory of COVID-19.

There are some other limitations to the present study that should be considered when interpreting the results. First, this study was a retrospective multicenter observational study; missing values of baseline serum biomarkers, especially IL-6, troponin, and BNP/NT-proBNP, which limited our further analysis. We did not unify the way of testing cardiac troponin (high-sense troponin or troponin) or BNP (BNP/NT-proBNP), which was also a limitation of our registry. In addition, as mentioned above, patients with a relatively stable clinical course were not transiently checked for biomarkers; therefore, there might have been a potential selection bias in interpreting the data. Furthermore, some values of biomarkers, especially for D-dimer, may not be comparable among the assays and manufacturers, although previous literature from Japan demonstrated that the current interassay imprecision is low [39,40]. Secondly, since this study targeted patients with COVID-19 and pre-existing CVD, the number of patients was relatively small. Further, it is unclear whether our results can be extrapolated to COVID-19 patients in other countries. In particular, the Japanese government mandated the hospitalization of all COVID-19 patients during patient enrollment; thus, the severity of hospitalized patients would be lower than that of other areas. A larger study with international collaboration is warranted for a robust evaluation of the relationship between biomarkers and COVID-19 outcomes. Finally, we did not evaluate the impact of treatment, especially for anticoagulation and steroids, which could confound the systemic immune response and transient biomarker levels [8]. However, despite these limitations, our study demonstrated that both CRP and D-dimer levels may be useful predictors for in-hospital mortality rates in COVID-19 patients with pre-existing CVD. In conclusion, our CLAVIS-COVID study revealed that the majority of patients had biomarker elevation representative of marked inflammation or hypercoagulopathy at hospital presentation in patients with COVID-19 and pre-existing CVD. The combination of these biomarkers may predict the incremental prognostic information for risk stratification and provide a framework for rapid risk estimation.

## Figures and Tables

**Table 1:** Baseline characteristics stratified by a combination of biomarkers.

Variables	Neither above Threshold (*n* = 71)	Either above Threshold (*n* = 148)	Both above Threshold (*n* = 242)	*p* Value
Demographics				
Age, years	62.0 ± 14.3	69.5 ± 16.3	70.6 ± 13.0	<0.001
Male, %	53.5	64.9	69.4	0.045
Japanese, %	94.4	98.8	98.8	0.079
BMI, kg/m^2^	25.2 ± 4.7	23.9 ± 4.9	23.9 ± 5.1	0.006
Comorbidities and medical history				
Hypertension, %	74.6	75.0	68.2	0.282
Diabetes mellitus, %	22.5	35.8	48.8	<0.001
Dyslipidemia, %	49.3	35.1	41.7	0.124
Heart failure, %	7.0	11.5	9.9	0.589
Coronary artery disease,%	7.0	11.5	9.5	0.573
Myocardial infarction, %	0.0	5.4	5.8	0.120
CI/TIA, %	4.2	6.8	10.3	0.188
COPD, %	4.2	3.4	7.4	0.206
CKD, %	5.6	7.4	9.5	0.528
Cancer, %	4.2	8.1	10.7	0.218
Symptoms				
Fever (>38.0 °C), %	55.1	61.9	69.5	0.062
Cough, %	47.9	45.3	53.7	0.248
Pharyngitis, %	16.9	14.2	8.7	0.087
Rhinorrhea, %	7.0	4.1	3.7	0.472
Dyspnea, %	15.5	33.8	47.5	<0.001
Arthritis, %	7.0	4.1	2.5	0.192
Headache, %	9.9	8.8	5.8	0.374
Olfactory dysfunction, %	9.9	10.1	5.4	0.167
Asymptom, %	9.9	9.5	3.7	0.038
4C Mortality Score	8 (6–11)	12 (9–14)	14 (11–15)	< 0.001
Physical findings				
Max body temperature	38.0 (37.5–38.3)	38.0 (37.6–38.5)	38.0 (37.7–38.6)	0.005
Herat rate (bpm)	85.0 (76.0–95.0)	82.0 (73.0–95.0)	88.0 (75.0–101.0)	0.028
Systolic BP (mmHg)	140.0 (119.0–154.0)	130.0 (117.0–150.0)	130.0 (115.0–142.5)	0.042
Respiratory rate (/min)	18.0 (16.0–22.0)	20.0 (17.0–24.0)	20.0 (18.0–25.3)	0.001
SpO2, %	97.0 (96.0–98.0)	96.0 (95.0–98.0)	95.0 (92.0–97.0)	<0.001
Laboratory data at admission				
White blood cell, /μL	4800 (3760–5900)	5400 (4400–7400)	6600 (5000–8800)	<0.001
Lymphocyte, %	24.0 (17.9–28.5)	18.3 (12.9–25.4)	12.3 (8.4–17.5)	<0.001
Neutrocyte, %	65.2 (60.7–73.4)	72.3 (63.8–80.7)	80.3 (73.7–86.2)	<0.001
Eosinocyte, %	0.50 (0.00–1.20)	0.30 (0.00–1.15)	0.00 (0.00–0.500)	<0.001
Hemoglobin, g/dl	14.0 (12.8–15.1)	13.4 (11.6–14.9)	13.0 (11.5–14.5)	0.003
Platelet, 10^3^/μL	184.0 (149.0–238.0)	178.0 (140.0–235.0)	196.0 (140.0–251.0)	0.657
Creatinin, mg/dL	0.77 (0.62–0.88)	0.85 (0.65–1.10)	0.87 (0.65–1.14)	0.012
eGFR, mL/min/1.73 m^2^	94.6 (77.4–105.5)	86.1 (64.1–106.6)	82.6 (60.3–109.2)	0.058
LDH, IU/L	219.0 (183.5–256.0)	276.5 (216.0–344.5)	380.0 (265.0–501.0)	<0.001
HbA1c, %	6.1 (5.9–6.8)	6.2 (5.7–6.8)	6.5 (6.1–7.4)	0.001
CK, U/L	73.5 (49.0–108.3)	84.5 (43.5–142.5)	88.0 (55.0–189.0)	0.089
Serum Alb, gL	4.0 (3.7–4.2)	3.4 (3.0–3.8)	3.0 (2.6–3.3)	<0.001
Specific biomarker at admission				
CRP, mg/L	7.8 (2.5–14.7)	30.5 (11.9–74.9)	106.0 (66.0–159.0)	<0.001
D-dimer, mg/L	0.60 (0.50–0.70)	0.90 (0.60–1.70)	2.24 (1.49–5.31)	<0.001
FDP, μg/mL	2.50 (1.60–2.95)	5.25 (3.42–6.40)	6.6 (5.0–12.9)	<0.001
Ferritin, ng/mL	234.0 (105.5–460.0)	476.0 (175.8–1000.8)	752.0 (312.0–1395.0)	<0.001
Procalcitonin, ng/mL	0.050 (0.020–0.750)	0.100 (0.058–0.193)	0.160 (0.080–0.570)	<0.001
KL-6, U/mL	277.0 (187.5–398.8)	276.0 (204.0–490.5)	332.0 (234.3–471.5)	0.071
BNP, pg/mL	11.0 (5.8–34.9)	29.1 (11.7–134.0)	58.9 (15.6–169.3)	0.001
Length of Stay, days	15.0 (11.0–25.0)	19.0 (13.0–27.8)	19.5 (10.0–32.0)	0.097

Data are shown as mean ± standard deviation or median with interquartile range or percentage. BMI: Body mass index, CI: Cerebral infarction, TIA: Transient ischemic attack, COPD: Chronic obstructive pulmonary disease, CKD: Chronic kidney disease, BP: Blood pressure, eGFR: Estimated glomerular filtration rate, LDH: Lactic acid dehydrogenase, HbA1c: Hemoglobin A1c, CK: Creatine kinase, Alb: Albumin, CRP: C-reactive protein, FDP: Fibrin degradation products, KL-6: Krebs von den Lungen-6 antigen, BNP: Brain natriuretic peptide.

**Table 2:** Independent predictor of in-hospital mortality.

Variables	HR	CI	*p* Value
Age	1.08	1.04–1.11	<0.000
Male	1.60	0.85–3.01	0.147
BMI	1.11	1.04–1.18	0.001
Hypertension	0.97	0.51–1.86	0.930
Diabetes mellitus	1.06	0.60–1.89	0.833
Dyslipidemia	0.94	0.52–1.68	0.831
Coronary artery disease	2.09	1.05–4.15	0.035
Cancer	1.26	0.60–2.62	0.541
COPD	2.56	1.23–5.34	0.012
CKD	1.28	0.60–2.75	0.524
Both values above threshold (CRP = 29.0 mg/L, D-dimer = 1.00 mg/L)	2.97	1.57–5.60	0.001

The Cox proportional hazard model predicting the odds of in-hospital mortality, adjusted for demographics and clinical comorbidities. Covariates included in the multivariable models were age, sex, body mass index (BMI), hypertension, diabetes mellitus, dyslipidemia, coronary artery disease, previous history of cancer, chronic obstructive pulmonary disease (COPD), chronic kidney disease (CKD), and baseline laboratory values. HR: Hazard ratio, CI: Confidence interval, BMI: Body mass index, COPD: Chronic obstructive pulmonary disease, CKD: Chronic kidney disease, CRP: C-reactive protein.

## Data Availability

The data presented in this study are available on request from the corresponding author.

## Supplementary Materials

The following are available online at https://www.mdpi.com/article/10.3390/jcm10143086/s1, Figure S1: Sensitivity analysis with additional threshold values to evaluate the incidence of in-hospital death, Figure S2: Proportion of patients whose peak CRP and D-dimer values were recorded at the indicated time points (days from admission), Figure S3: Trajectories of other biomarkers over time among survival and in-hospital deaths, Table S1: Assays and Manufactures for C-Reactive Protein and D-dimer Measurements, Table S2: Univariate cox proportional hazard analysis for predictor of in-hospital mortality, Table S3: Multivariate cox proportional hazard analysis for predictor of in-hospital mortality, Table S4: Biomarker levels at each timing of admission, peak, and final examination, Table S5: The increment of biomarker values between baseline and peak, Table S6: Timing of peak value of each biomarker (Days from discharge).
