# Supplementary material for: Inflammatory and Hypercoagulable Biomarkers and Clinical Outcomes in COVID-19 Patients
Source: J Clin Med. 2021 Jul 13;10(14):3086. doi: 10.3390/jcm10143086 (PMC8304719; doi:10.3390/jcm10143086)
Supplement: Supplementary file 1 [file jcm-10-03086-s001.zip › jcm-1281701-supplementary.pdf]

**Supplemental material**

**Number of Supplemental Table: 6 Tables**

**Number of Supplemental Figure: 3 Figures**

**Table S1A. Assays and Manufactures for C-Reactive Protein Measurements**

| Assays | Manufactures |
|---|---|
| Latex agglutination | EIKEN CHEMICAL CO.,LTD. |
| | SEKISUI MEDICAL CO., LTD. |
| | NITTOBO MEDICAL CO.,LTD. |
| | FUJIFILM Wako Pure Chemical Corporation |
| | LSI Medience Corporation. |
| Latex turbidimetry | Shino-Test Corporation. |
| | LSI Medience Corporation. |
| | Roche Diagnostics K.K. |

**Table S1B. Assays and Manufactures D-dimer Measurements**

| Assays | Manufactures |
|---|---|
| Latex agglutination | LSI Medience Corporation. |
| | bioMérieux Japan Ltd. |
| | Roche Diagnostics K.K. |
| Latex turbidimetry | LSI Medience Corporation. |
| | bioMérieux Japan Ltd. |
| | Roche Diagnostics K.K. |
| | Sysmex Corporation. |
| | SEKISUI MEDICAL CO., LTD. |
| | KAINOS Laboratories, Inc. |
| | KYOKUTO PHARMACEUTICAL INDUSTRIAL CO., LTD. |

**Table S2. Univariate cox proportional hazard analysis for predictor of in-hospital mortality**

| Variables | HR | CI | | p |
|---|---|---|---|---|
| Demographics and medical history | | | | |
| Age | 1.05 | 1.03 | 1.07 | <0.001 |
| Male | 1.48 | 0.88 | 2.47 | 0.136 |
| Japanese | 21.10 | 0.06 | 7995.92 | 0.314 |
| BMI | 1.00 | 0.94 | 1.05 | 0.885 |
| Comorbidities | | | | |
| Hypertension | 1.16 | 0.70 | 1.92 | 0.571 |
| Diabetes mellitus | 0.81 | 0.51 | 1.30 | 0.388 |
| Dyslipidemia | 0.73 | 0.45 | 1.18 | 0.198 |
| Heart failure | 1.59 | 0.85 | 2.94 | 0.145 |
| Coronary artery disease | 2.41 | 1.36 | 4.24 | 0.002 |
| Myocardial infarction | 1.58 | 0.72 | 3.46 | 0.252 |
| CI/TIA | 0.96 | 0.47 | 1.95 | 0.913 |
| Chronic lung disease | 3.11 | 1.63 | 5.91 | 0.001 |
| CKD | 1.73 | 0.93 | 3.21 | 0.084 |
| Cancer | 2.53 | 1.41 | 4.56 | 0.002 |
| Symptoms | | | | |
| Fever (> 38.0°C) | 1.67 | 0.95 | 2.92 | 0.076 |
| Cough | 1.10 | 0.70 | 1.74 | 0.668 |
| Pharyngitis | 0.48 | 0.17 | 1.34 | 0.160 |
| Rhinorrhea | 0.86 | 0.26 | 2.84 | 0.801 |
| Dyspnea | 1.50 | 0.95 | 2.36 | 0.080 |
| Arthritis | 1.16 | 0.37 | 3.70 | 0.798 |
| Headache | 0.05 | 0.00 | 2.82 | 0.142 |
| Olfactory dysfunction | 0.05 | 0.00 | 4.02 | 0.176 |
| Asymptom | 0.56 | 0.18 | 1.79 | 0.330 |
| Physical findings | | | | |
| Max body temperature | 1.38 | 1.04 | 1.84 | 0.027 |
| Herat rate | 1.02 | 1.00 | 1.03 | 0.017 |
| Systolic BP | 0.99 | 0.99 | 1.00 | 0.249 |
| Respiratory rate | 1.06 | 1.02 | 1.11 | 0.002 |
| SpO2 | 0.95 | 0.92 | 0.98 | 0.003 |
| Laboratory data at admission | | | | |
| White blood cell | 1.00 | 1.00 | 1.00 | 0.050 |
| Lymphocyte | 0.96 | 0.94 | 0.99 | 0.015 |

| | | | | |
|---|---|---|---|---|
| Neutrocyte | 1.03 | 1.01 | 1.05 | 0.019 |
| Eosinocyte | 0.70 | 0.50 | 0.98 | 0.038 |
| Hemoglobin | 0.92 | 0.83 | 1.02 | 0.096 |
| Platelet | 0.95 | 0.91 | 0.98 | 0.001 |
| Creatinin | 1.14 | 1.04 | 1.24 | 0.003 |
| eGFR | 0.99 | 0.98 | 1.00 | 0.002 |
| LDH | 1.00 | 1.00 | 1.00 | <0.001 |
| HbA1c | 0.80 | 0.60 | 1.07 | 0.137 |
| CK | 1.00 | 1.00 | 1.00 | 0.689 |
| Serum Alb | 0.51 | 0.36 | 0.73 | <0.001 |
| Specific biomarker at admission | | | | |
| CRP | 1.03 | 1.00 | 1.05 | 0.029 |
| D-dimer | 1.00 | 1.00 | 1.01 | 0.341 |
| FDP | 1.00 | 1.00 | 1.00 | 0.989 |
| Ferritin | 1.00 | 1.00 | 1.00 | 0.602 |
| Procalcitonin | 1.00 | 0.98 | 1.03 | 0.725 |
| IL-6 | 1.00 | 0.99 | 1.01 | 0.944 |
| KL-6 | 1.00 | 1.00 | 1.00 | 0.001 |
| Both CRP and D-dimer were above Middle | 3.46 | 1.72 | 6.96 | <0.001 |

Data are shown as mean ± standard deviation or median with interquartile range or percentage. Variables of laboratory data were analyzed as continuous variables, except for cumulative variable of CRP and D-dimer. BMI: body mass index, CI: cerebral infarction, TIA: transient ischemic attack, COPD: chronic obstructive pulmonary disease, CKD: chronic kidney disease, BP: blood pressure, eGFR: estimated glomerular filtration rate, LDH: lactic acid dehydrogenase, HbA1c: hemoglobin A1c, CK: creatine kinase, Alb: albumin, CRP: C-reactive protein, FDP: fibrin degradation products, KL-6: Krebs von den Lungen-6 antigen.

**Table S3. Multivariate cox proportional hazard analysis for predictor of in-hospital mortality**

| Variables | HR | CI | | p |
|---|---|---|---|---|
| Demographics and medical history | | | | |
| Age | 1.07 | 1.023 | 1.111 | 0.003 |
| Coronary artery disease | 3.39 | 1.358 | 8.445 | 0.009 |
| COPD | 1.18 | 0.353 | 3.917 | 0.793 |
| CKD | 1.92 | 0.592 | 6.195 | 0.278 |
| Cancer | 0.66 | 0.213 | 2.042 | 0.470 |
| Dyspnea | 0.85 | 0.360 | 1.989 | 0.702 |
| Max body temperature | 1.56 | 0.980 | 2.472 | 0.061 |
| Herat rate | 1.02 | 0.993 | 1.039 | 0.183 |
| SpO2 | 0.96 | 0.899 | 1.034 | 0.303 |
| White blood cell | 1.00 | 1.000 | 1.000 | 0.944 |
| Hemoglobin | 1.04 | 0.829 | 1.299 | 0.748 |
| Platelet | 0.92 | 0.857 | 0.992 | 0.030 |
| LDH | 1.00 | 0.998 | 1.004 | 0.521 |
| Serum Alb | 0.83 | 0.315 | 2.189 | 0.706 |
| KL-6 | 1.00 | 1.000 | 1.002 | 0.181 |
| Both CRP and D-dimer were above Middle | 3.88 | 1.185 | 12.728 | 0.025 |

The Cox proportional hazard model predicting the odds of in-hospital mortality, adjusted for demographics and clinical comorbidities. Variables of laboratory data were analyzed as continuous variables, except for cumulative variable of CRP and D-dimer. Covariates included in the multivariable were selected based on univariate analyses.

HR: hazard ratio, CI: confidence interval, BMI: body mass index, COPD: chronic obstructive pulmonary disease, CKD: chronic kidney disease, LDH: lactic acid dehydrogenase, Alb: albumin, KL-6: Krebs von den Lungen-6 antigen, CRP: C-reactive protein.

**Table S4. Biomarker levels at each timing of admission, peak, and final examination**

| | In-hospital death (*n*=108) | *n* | Non in-hospital death (*n*=585) | *n* | *P* Value |
|---|---|---|---|---|---|
| CRP, mg/L | | | | | |
| At admission | 107.2 (56.9 – 168.9) | 108 | 49.6 (13.4 – 104.5) | 559 | <0.001 |
| Peak | 247.6 (153.3 – 326.7) | 104 | 816.2 (301.5 – 1524.3) | 544 | <0.001 |
| Final | 156.0 (748.5 – 2374.5) | 92 | 4.7 (1.4 – 17.5) | 484 | <0.001 |
| D-dimer, mg/L | | | | | |
| At admission | 2.15 (1.32 - 5.08) | 76 | 1.30 (0.70 - 2.60) | 385 | <0.001 |
| Peak | 14.1 (3.7 - 51.9) | 82 | 2.40 (1.00 - 7.12) | 403 | <0.001 |
| Final | 14.2 (5.93 - 36.1) | 58 | 1.90 (1.01 - 4.00) | 239 | <0.001 |

CRP: C-reactive protein.

**Table S5. The increment of biomarker values between baseline and peak**

| | In-hospital death (*n*=108) | *n* | Non in-hospital death (*n*=585) | *n* | *P* Value |
|---|---|---|---|---|---|
| CRP, mg/L | 117.1 (15.1 – 209.2) | 104 | 0.15 (0.00 – 52.3) | 544 | <0.001 |
| D-dimer, mg/L | 6.05 (0.00 - 31.1) | 74 | 0.00 (0.00 - 2.69) | 376 | <0.001 |
| FDP, µg/mL | 1.3 (0.00 - 50.0) | 50 | 0.00 (0.00 - 2.90) | 158 | 0.004 |
| Ferritin, ng/mL | 29.5 (0.00 - 750.0) | 40 | 0.00 (0.00 - 15.0) | 271 | 0.001 |
| Procalcitonin, ng/mL | 0.22 (0.00 - 2.14) | 52 | 0.00 (0.00 - 0.00) | 205 | <0.001 |
| KL6, U/mL | 0.00 (0.00 - 269.0) | 46 | 0.00 (0.00 - 0.00) | 240 | 0.002 |

CRP: C-reactive protein, FDP: fibrin degradation products, KL-6: Krebs von den Lungen-6 antigen.

**Table S6. Timing of peak value of each biomarker (Days from discharge)**

| | In-hospital death (*n*=108) | *n* | Non in-hospital death (*n*=585) | *n* | *P* Value |
|---|---|---|---|---|---|
| CRP | 4.5 (0.25 - 14.0) | 104 | 15.0 (9.0 - 24.0) | 546 | <0.001 |
| D-dimer | 5.0 (1.0 - 13.0) | 82 | 13.5 (8.0 - 23.8) | 404 | <0.001 |
| FDP | 8.0 (1.0 - 22.0 ) | 59 | 16.0 (9.0 - 28.0) | 179 | 0.001 |
| Ferritin | 7.0 (1.0 - 19.0) | 49 | 15.0 (9.8 - 25.3) | 302 | <0.001 |
| Procalcitonin | 8.0 (2.0 - 23.0 ) | 55 | 17.0 (11.0 - 24.0) | 222 | 0.001 |
| KL6 | 8.5 (5.3 - 18.8) | 48 | 14.0 (8.0 - 25.0) | 267 | 0.012 |

CRP: C-reactive protein, FDP: fibrin degradation products, KL-6: Krebs von den Lungen-6 antigen.

# Figure S1

## (A)

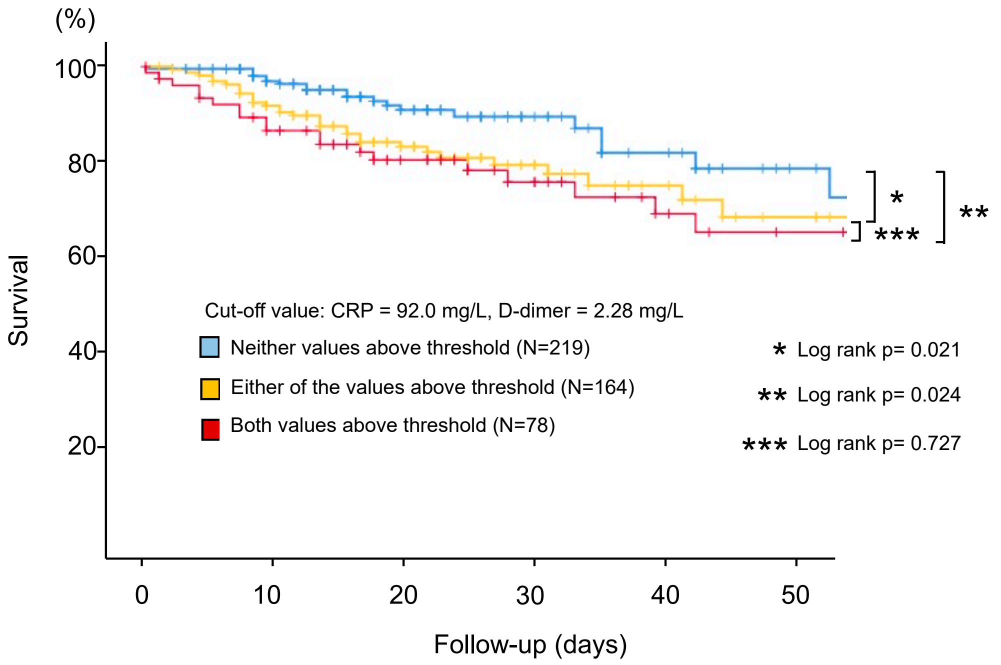
(%)
100
80
60
40
20
Survival
0
10
20
30
40
50
Follow-up (days)
Cut-off value: CRP = 92.0 mg/L, D-dimer = 2.28 mg/L
Neither values above threshold (N=219)
Either of the values above threshold (N=164)
Both values above threshold (N=78)
* Log rank p= 0.021
** Log rank p= 0.024
*** Log rank p= 0.727
*
**
***

## (B)

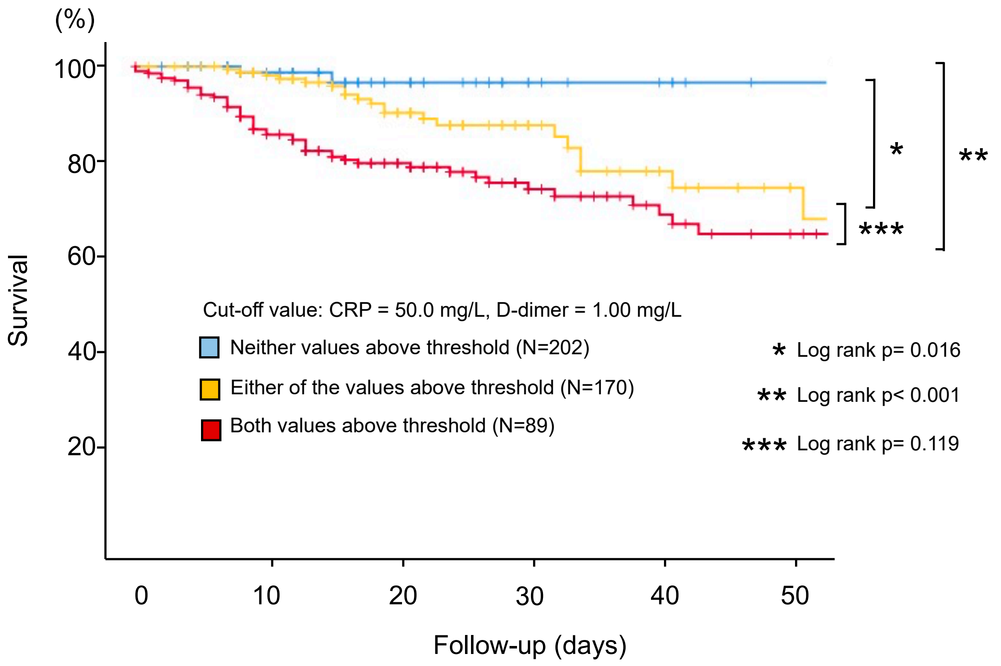
(%)
100
80
60
40
20
Survival
0
10
20
30
40
50
Follow-up (days)
Cut-off value: CRP = 50.0 mg/L, D-dimer = 1.00 mg/L
Neither values above threshold (N=202)
Either of the values above threshold (N=170)
Both values above threshold (N=89)
* Log rank p= 0.016
** Log rank p< 0.001
*** Log rank p= 0.119
*
**
***

# Figure S2

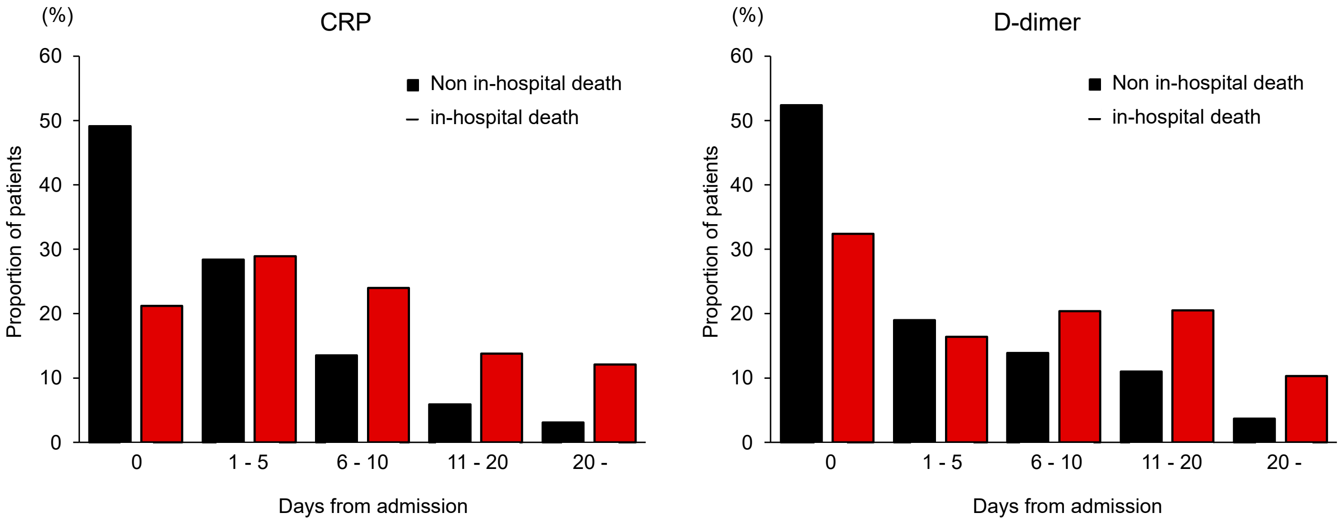
CRP
(%)
60
50
40
30
20
10
0
Proportion of patients
Non in-hospital death
in-hospital death
0
1 - 5
6 - 10
11 - 20
20 -
Days from admission
D-dimer
(%)
60
50
40
30
20
10
0
Proportion of patients
Non in-hospital death
in-hospital death
0
1 - 5
6 - 10
11 - 20
20 -
Days from admission

# Figure S3

## (A)

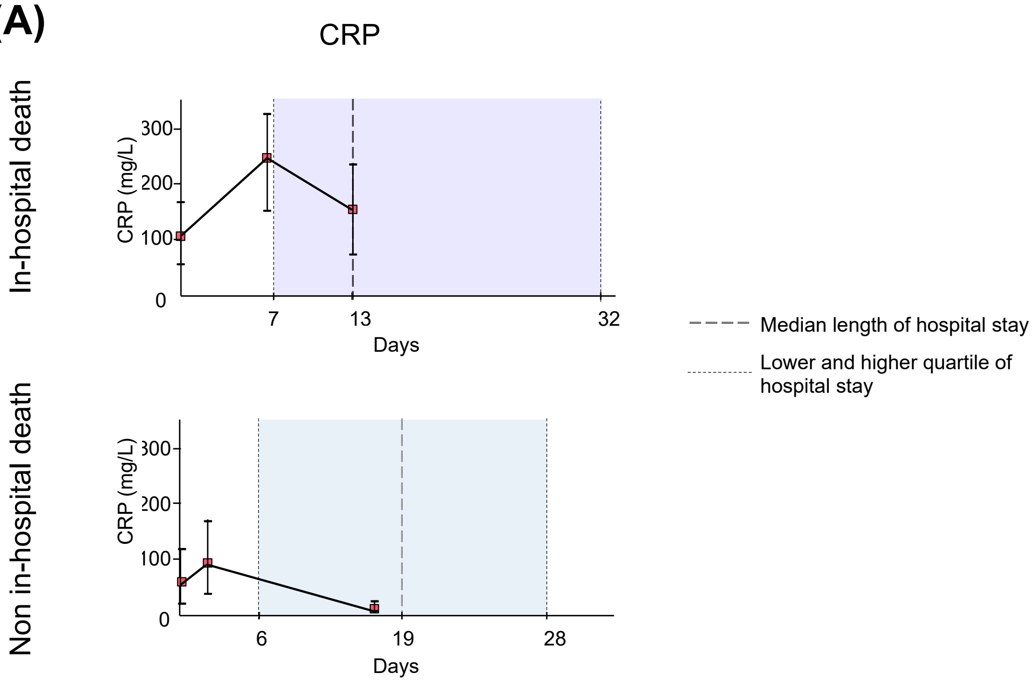
CRP
In-hospital death
Non in-hospital death
CRP (mg/L)
0
100
200
300
7
13
32
6
19
28
Days
Median length of hospital stay
Lower and higher quartile of hospital stay

## (B)

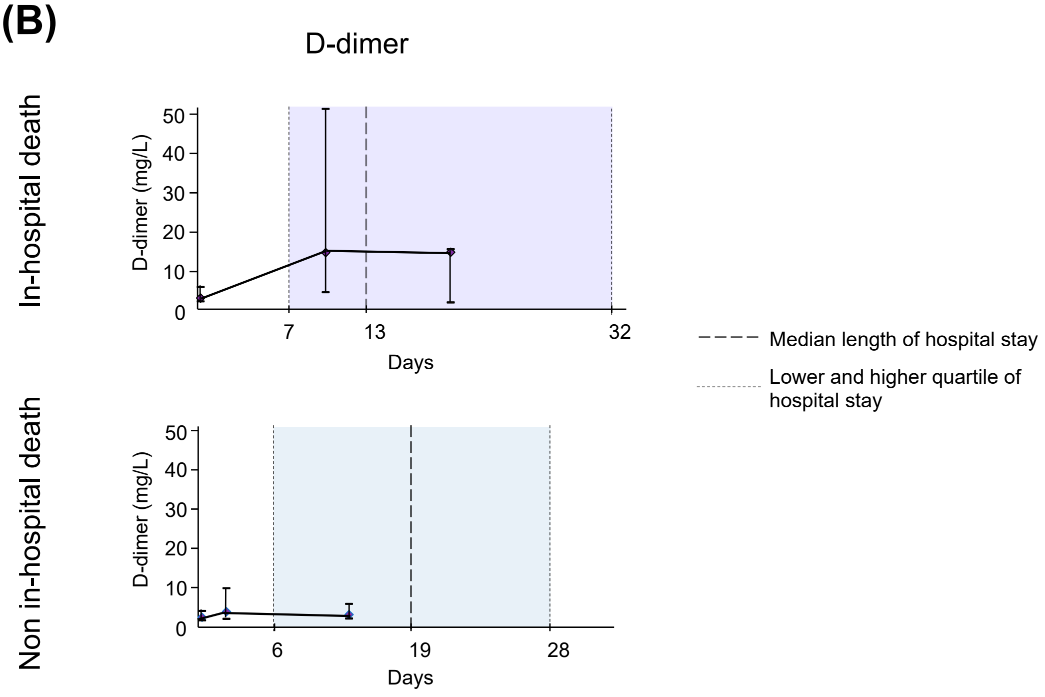
D-dimer
In-hospital death
Non in-hospital death
D-dimer (mg/L)
0
10
20
30
40
50
7
13
32
6
19
28
Days
Median length of hospital stay
Lower and higher quartile of hospital stay

**(C)**

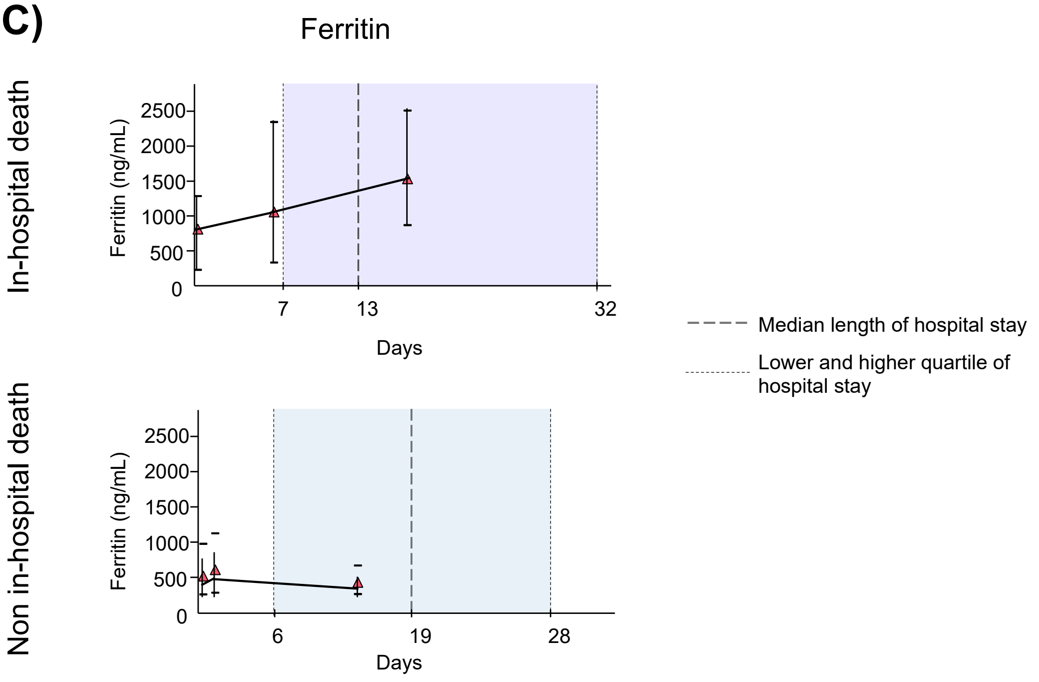
Ferritin
In-hospital death
Ferritin (ng/mL)
2500
2000
1500
1000
500
0
7
13
32
Days
Median length of hospital stay
Lower and higher quartile of hospital stay
Non in-hospital death
Ferritin (ng/mL)
2500
2000
1500
1000
500
0
6
19
28
Days

**(D)**

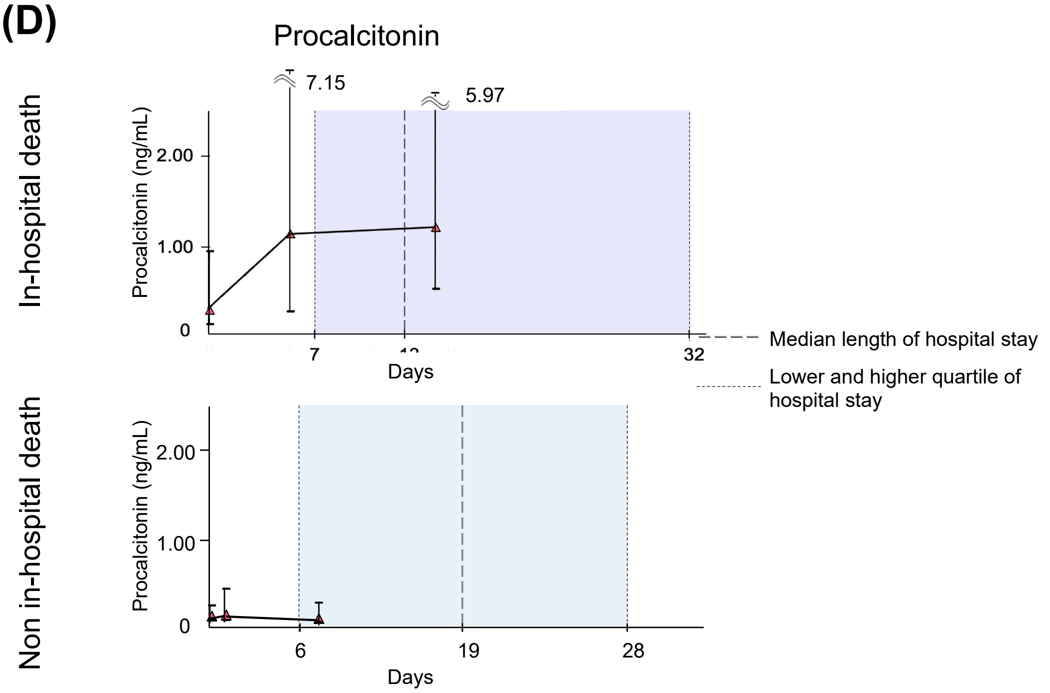
Procalcitonin
7.15
5.97
In-hospital death
Procalcitonin (ng/mL)
2.00
1.00
0
7
32
Days
Median length of hospital stay
Lower and higher quartile of hospital stay
Non in-hospital death
Procalcitonin (ng/mL)
2.00
1.00
0
6
19
28
Days

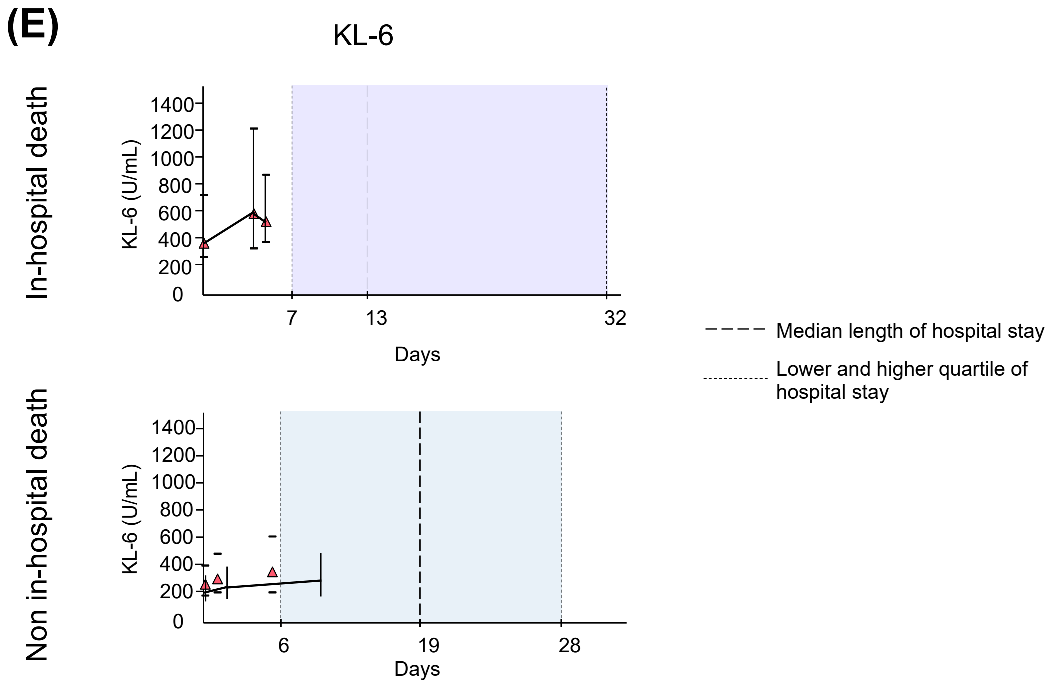
(E)
KL-6
In-hospital death
Non in-hospital death
KL-6 (U/mL)
1400
1200
1000
800
600
400
200
0
7
13
32
6
19
28
Days
Median length of hospital stay
Lower and higher quartile of hospital stay

## Supplemental Figure Legend

Figure S1: Sensitivity analysis with additional threshold values to evaluate the incidence of in-hospital death. Threshold values were: (A) CRP, 92.0 mg/L; D-dimer, 2.28 mg/L, and (B) CRP, 50.0 mg/L; D-dimer, 1.00 mg/L. CRP: C-reactive protein. CRP: C-reactive protein.

Figure S2: Proportion of patients whose peak CRP and D-dimer values were recorded at the indicated time points (days from admission). CRP: C-reactive protein.

Figure S3: Trajectories of other biomarkers over time among survival and in-hospital deaths. (A) CRP, (B) D-dimer, (C) ferritin, (D) procalcitonin, and (E) KL-6. CRP: C-reactive protein.
